# Antioxidative Status and Meat Quality Traits as Affected by Dietary Supplementation of Finishing Pigs with Natural Phenolics

**DOI:** 10.3390/antiox13111362

**Published:** 2024-11-07

**Authors:** Małgorzata Muzolf-Panek, Anita Zaworska-Zakrzewska, Anna Czech, Dariusz Lisiak, Małgorzata Kasprowicz-Potocka

**Affiliations:** 1 Department of Food Quality and Safety Management, Faculty of Food Science and Nutrition, Poznań University of Life Sciences, Wojska Polskiego 31, 60-637 Poznań, Poland; 2 Department of Animal Nutrition, Faculty of Veterinary Medicine and Animal Science, Poznań University of Life Sciences, Wołyńska 33, 60-637 Poznań, Poland; malgorzata.potocka@up.poznan.pl; 3 Department of Biochemistry and Toxicology, University of Life Sciences in Lublin, Akademicka 13, 20-950 Lublin, Poland; anna.czech@up.lublin.pl; 4 Department of Primary Meat Production, Institute of Agricultural and Food Biotechnology—State Research Institute, Głogowska 239, 60-111 Poznań, Poland; dariusz.lisiak@ibprs.pl

**Keywords:** phenolic supplementation, pork quality, antioxidant activity, color, oxidative stability, catalase, superoxide dismutase

## Abstract

This work investigated the effect of a plant-based phenolic supplement on the color, myoglobin forms, lipid oxidation, and antioxidative status of fresh and stored (10 days at 4 °C) meat (*Longissimus thoracis et lumborum*), as well as the antioxidative status of the blood and liver. The sensory quality of the meat was also evaluated for color, aroma, texture, juiciness, and palatability. Twenty-four finishing pigs, divided into two groups, were fed a basal diet and a diet with a phenolic supplement (0.1%). The supplementation increased the redness of the meat (+36% for a* and +28% for redness index), the myoglobin (Mb) content (+7%), the antioxidant activity, and the juiciness. The treatment increased the antioxidant status of meat, reflected by superoxide dismutase (SOD) activity and total glutathione (GSH + GSSG). The catalase and SOD activities and GSH + GSSG of the blood and liver were also elevated in the supplemented samples when compared to the control group. A significant effect of time was observed for all tested parameters (pH, color attributes, Mb forms, the antioxidant activity, lipid oxidation) except for the Mb content. For the stored samples, only TBARSs (thiobarbituric acid reagent substances) were affected by the diet. The slope of the plot for TBARS changes with time was significantly different between the control and treated groups (*p* = 0.017), which indicated a significant effect of dietary supplementation. A higher rate of lipid oxidation was observed in the control samples.

## 1. Introduction

Nowadays, pork is one of the most widely consumed meat types in the world (35%) after poultry (39%), followed by beef (21%) and lamb (5%), as indicated by the United Nations Food and Agriculture Organization [1]. According to an OECD-FAO report, worldwide pig meat production reached 117 million tons of carcass weight in 2022 and is projected to increase by 11% to 129 million tons in 2032. Total pork meat consumption is also expected to grow by 11% reaching 93 million tons of retail weight in 2032 and is expected to become the second largest contributor to the total growth in meat consumption [1]. The constantly increasing demand for animal protein is leading producers to continuously improve feed efficiency, while lowering feed intakes and reducing costs, which is in line with the “Farm to Fork” strategy, pointing at sustainable food production [1]. However, faster growth of muscle tissues is often associated with the oxidative stress that affects meat quality. Thus, ensuring and maintaining a high quality of meat are key elements for both producers and consumers. The oxidative stress is induced in the living organism by the reactive oxygen species (ROS) and becomes, itself, a source of ROS that initiates the oxidation of muscle components and, thus, meat.

Lipid oxidation is the major cause of the quality decrease and reduced shelf life of meat products [2]. As an effect of lipid oxidation, primary products like alkyl, alkoxy, or peroxy radicals are produced; they may be ready for further reaction with other molecules. When oxidation proceeds, lipid peroxides could undergo scission to form secondary products, including aldehydes, ketones, and epoxides [3,4]. This process limits the acceptability of meat and meat products and negatively affects the sensory attributes, such as color, taste, odor, and nutritional value, due to a reduction in the fat-soluble vitamin content, changes in fatty acid composition, and, finally, food safety, such as the formation of toxic compounds [2,5,6,7]. Other meat components like pigments or proteins also undergo oxidative changes [8,9,10,11,12,13,14,15,16].

Among meat proteins, myoglobin is a heme pigment in muscle cells that influences mostly the meat sensory quality and, thus, the product consumers’ assessment as the meat discoloration proceeds with the pigment oxidative modification [3,12,17,18]. Myoglobin consists of a globin chain and a heme iron center positioned in the hydrophobic pocket [19]. Generally, the oxidation of myoglobin refers to the oxidation of iron in the heme group [17]. Myoglobin in meat is present in three major redox forms depending on the iron valent state and the presence of a ligand at the sixth site of the iron atom, which are responsible for meat color. The three forms are a dark purplish-red/pink dexoymyoglobin—DeoMb; a bright-red oxymyoglobin—OxyMb; and a tan to brown metmyoglobin—MetMb [19,20].

The route of the interconvertion of myoglobin redox forms in fresh meat depends on oxygen availability, pH, and temperature [19].

Many factors affect the oxidative stability of meat, including husbandry (breed, diet, muscles, etc.) and peri- and post-mortem factors (animal handling, transport, slaughtering, meat chilling and ageing, technological processes, packaging, and storage) [18,21].

Muscle tissue contains several endogenous enzymatic and non-enzymatic antioxidants, namely, enzymes, such as catalase, superoxide dismutase, glutathione peroxidase, or low-weight molecules like glutathione, which maintain redox balance in the organism. They also act as a post-mortem in meat to counteract and/or inhibit the oxidative stress induced during biochemical changes in muscles into meat. However, their activity decreases when oxidation proceeds [9]. To maintain meat quality during shelf-life and to increase its oxidative stability, various strategies have been applied.

An effective method of mitigating the effect of oxidation in fresh raw meat and maintaining its nutritional quality during handling is the use of antioxidants. Antioxidants can be applied in various manners, either directly through the addition to meat or indirectly as the result of nutritional interventions to animals. The active packaging with antioxidant active coatings is also extensively tested [22,23]. Plants or extracts of various plants are good sources of natural antioxidants [24], and, generally, their addition to food meets consumers’ acceptance since they are found to be nontoxic [25,26]. Many studies and reviews have shown the beneficial effects of natural antioxidants in meat and meat products [2,4,23,27,28]. However, the exogenous incorporation of some natural antioxidants into meat could affect its color [4], which is a key attribute of consumers’ sensory assessment and could affect consumers’ decisions at purchase [26]. Moreover, the strategy is dedicated mostly to minced meat and not all retail cuts [25]. The second way to maintain meat quality and/or inhibit oxidation processes during handling is the endogenous application of antioxidants, i.e., the supplementation of animals’ diets with antioxidants. It was previously shown that an animal diet supplemented with bioactive compounds could affect meat quality, like meat color [29], lipid and protein oxidation [30,31,32], fatty acid content [30,31,33], or enzymatic and non-enzymatic endogenous antioxidants [29,32,34,35]. The phytochemicals in tested extracts are phenolic compounds, namely, proanthocyanidins in grape seeds [29,31], flavonoids and other polyphenols in citrus by-products [30] and mulberry leaves [34], or tocopherols, carotene, and lycopene in tomatoes [33]. Phenolic supplementation could also affect the meat productivity by increasing growth performance [32]. It has also been reported that dietary supplementation with antioxidants has no effect on growth performance [34,36], meat quality parameters, including the oxidative stability of lipids [33,37] and proteins [38], color [30,33,37], and myoglobin forms [33].

A number of plant-based supplements for animal nutrition are available on the market, designed to increase or maintain the feed efficiency and, at the same time, to counteract the oxidative stress in meat [39,40]. They are claimed to contain a mixture of the various antioxidant active compounds, including tocopherols, phenolic acids, and polyphenols, and to improve meat quality by inhibiting or reducing oxidative stress [36,39,40]. The transfer of these bioactive compounds into meat tissue is hypothesized to improve the antioxidant properties of meat and increase its oxidative stability [41].

Taking the above into account, the aim of this study was to determine the quality parameters of raw fresh meat (*Longissimus thoracis et lumborum*), namely color, myoglobin forms, lipid oxidation, and the antioxidative status in order to provide evidence for the use of a phenolic-rich supplement to the finishing diet of pigs as the source of the antioxidant active compounds. To this end, the antioxidant activity of the plant-based supplement was also tested. Additionally, to better show the effect of the diet on meat quality changes, parameters such as color, lipid oxidation, and myoglobin forms were determined during refrigerated storage (10 days at 4 °C).

## 2. Materials and Methods

### 2.1. Chemicals

2,2′-azinobis-(3-ethylbenzothiazoline-6-sulfonic acid) (ABTS), 1,1′-diphenyl-2-picrylhydrazyl (DPPH), Folin-Ciocalteu phenol reagent, gallic acid (GA), quercetin (Q), 6-hydroxy-2,5,7,8-tetramethylchroman-2-carboxylic acid (Trolox), 2-thiobarbituric acid (TBA), 1,1,3,3-tetramethoxypropane and organic solvents of HPLC grade were supplied from Sigma-Aldrich (Steinheim, Germany). All other chemicals were of analytical grade and collected from POCh (Gliwice, Poland).

### 2.2. Animal Handling, Experimental Design, Diets and Sampling

The experiment was conducted on a local individual farm in Wielkopolska Voivodeship, Poland. Twenty-four female piglets with an initial body weight of about 31 kg were individually penned indoors and randomly assigned into 2 groups, each of twelve animals (n = 12). Each pig was individually identified with a numbered ear tag. The experiment lasted 100 days and was divided into two stages: grower (48 days) and finisher (52 days). The raw material composition and chemical composition of the basal diet are shown in Table 1.

The control group received no supplementation during the whole experiment, while in the experimental group (P-suppl.), 0.1% wheat cereals were replaced (1 kg per ton) by the composition of the natural phenolic supplement described in patent no WO2020254391A1 [42]. The supplement is evaluated in the Section 2.5. The complete diets were formulated according to the recommendations of Gesellschaft für Ernährungsphysiologie [43]. The pigs had unlimited access to feed and water.

The health and welfare of the animals were monitored during the experiment. At the end of the experiment, all (24 females) were stunned by electric shock and killed via exsanguination in a commercial slaughter. The blood samples were taken during exsanguination and directly collected into Vacutainer Serum Separator Tubes (BD SST II Advance, Franklin Lakes, NJ, USA). The samples were incubated for 15 min at room temperature to clot, and then they were centrifuged for 10 min at 3500× *g* at 4 °C. Serum was transferred into new tubes, immediately frozen, and stored at −80 °C until future analysis. The liver samples were collected on a slaughter line. A fragment of the tissue was cut off from the right upper lobe of the liver, put into an Eppendorf tube (Eppendorf Tubes^®^, UltraCruz^®^, Merck, Darmstadt, Germany), immediately frozen (dry ice-Solid Carbon Dioxide), and stored at −80 °C until analysis. The carcass was subjected to a moderate, conventional chilling regime (1–4 °C; air velocity 0.5 m/s), which allowed for rapid chilling (−5 °C) within 2 h, measured in the coolers (160 cm above the floor of the cooler), where the carcass is usually placed. The *Longissimus lumborum* along the first nine vertebrae of the right carcass side was obtained 24 h after slaughter. The meat samples (about 2 kg) were packed into the polypropylene bags (CRYOVAC^®^ packaging-safe bags, Cryovac Inc., Elmwood Park, NJ, USA, especially for food thickness < 100 µm) and transported within 1.5 h to the laboratory at 4 °C. The samples were stored in an ST 1/11 storage cabinet (Pol-Eko, Wodzisła Śląski, Poland) at 4 °C in the dark until the next day, except for meat intended for the antioxidant status analysis, for which the samples were immediately frozen and stored at −80 °C (dry ice).

### 2.3. Ethical Statement

Pigs were kept in accordance with the guidelines of the European Union Directive (2010/63/EU). All procedures were reviewed with the guidelines of the Local Ethical Committee for Experiments on Animals in Poznań regarding animal experimentation and the care of animals, but individual approval for this trial was not required because of the commercial production standards used in this study. All biological samples were collected after slaughter.

### 2.4. Feed Analyses

The feed mixtures used in the experiment were analyzed chemically twice (n = 2). A Retsch Zm 200 ultra-centrifugal mill (Retsch, Haan, Germany) with 1.0 mm sieves was used to grind the feed material. The material was analyzed for crude protein, crude fat, crude fiber, crude ash, dry matter, and total Ca and P. Methods 976.05, 942.05, 97.1, 2003.05, 935.13, and 965.17 were, respectively, used according to [44,45]. The determination of amino acids (lysine—Lys and methionine—Met) was performed according to the methodology described by Szkudzińska et al. [46].

### 2.5. Antioxidant Activity and Phenolic Compound Content of the Supplement

Next, 0.25 g of the plant-based natural supplement with polyphenols (P-suppl.) was extracted with 40 mL of 25% aqueous methanol for 30 min at ambient temperature on the magnetic stirrer (MS–H–Pro plus, Chemland, Stargard, Poland) with electronic control for constant speed. Then, after filtration through paper filters (type 388 filter, Filtrak, Niederschlag Bärenstein, Germany), the supernatant was collected and used for further analyses. Three separate extractions were performed.

The antioxidant activity of the sample was determined using three different methods, namely the DPPH, TEAC (Trolox equivalent antioxidant capacity), and FRAP (ferric-reducing antioxidant power).

The DPPH method was performed according to the procedure by Sànchez-Moreno et al. [47]. Briefly, the 0.1 mmol DPPH radical was generated in methanol and mixed with the extract in a ratio of 100:1. After 30 min of incubation in the dark at room temperature, the absorbance readings were collected at 515 nm (spectrophotometer Cary 1E, Varian, Belrose, Australia) using methanol for zero instrument calibration.

The TEAC method is based on the scavenging of ABTS^+•^ cation radicals by the antioxidants [48,49]. The ABTS^+•^ radical cation was generated with potassium disulphate, as indicated previously [48]. The absorbance of 100-times-diluted extract in 4% ABTS^+•^ in PBS (phosphate buffer saline, pH 7.4) was measured at 734 nm after 6 min of incubation in the dark. PBS was used for zero calibration of the instrument.

The FRAP assay was provided by Benzie and Strain [50]. The extract was mixed with the ferric tripyridyltriazine complex, and, after 15 min incubation at 37 °C, the absorbance was read at 593 nm. Acetic buffer was used to zero the spectrophotometer.

All results of the antioxidant activity measurements were expressed as TEAC values in µM Trolox/g supplement.

Total phenolic content (TPC) was determined spectrophotometrically based on the absorbance reading at 765 nm. The procedure of sample preparation was performed according to Singleton and Rossi [51] and described in detail by Muzolf-Panek et al. [24]. The results were expressed as mg gallic acid equivalent (GAE) per g of the supplement.

Total flavonoid content (TFC) was measured at 410 nm using the aluminum chloride method [52] described in our previous study [24]. Results were expressed as mg quercetin equivalent (QE) per g of supplement.

### 2.6. Meat Quality Determination

The analyses of fresh meat were conducted 48 h post-mortem. They included nutrient composition, water holding capacity, cooking loss, drip loss, cutting force (first three vertebrae), pH, color, myoglobin (Mb) content, myoglobin forms, the antioxidant activity, lipid oxidation, and sensory analysis (six vertebras). The color of meat 48 h post-mortem was measured just after cutting.

Measurements of pH after 45 min of slaughter (pH 45 min) and pH after 24 h of slaughter (pH 24 h) were performed to diagnose, with high probability, meat with the normal course of glycolysis (RFN—red, firm, normal meat) obtaining high-quality characteristics and to separate it from meat showing quality abnormalities of PSE (pale, soft exudative meat) or DFD (dark, firm, dry meat) types.

To monitor the meat quality during storage, the meat samples were divided into five 3 cm thick steaks (48 h after slaughter), each packed into 0.07 mm thick polypropylene bags (CRYOVAC^®^ packaging-safe bags) and stored in a ST 1/11 storage cabinet (Pol-Eko, Wodzisła Śląski, Poland) at 4 °C in the dark for 10 days. The analyses, namely pH, color, the Mb content, proportion of myoglobin forms, the antioxidant activity, and lipid oxidation, were performed on days 1, 4, 7, and 10 of storage, taking 48 h post-mortem as the starting point (coded as day 1 of storage). The same chops were analyzed for color, reflectance spectra, and pH at repetitive times. Other measurements (lipid oxidation, the antioxidant activity, and the Mb content) were made on one of the four prepared chops at given specific time points (days 1, 4, 7, and 10).

#### 2.6.1. Basic Chemical Composition

To determine the water content of meat, the drying method at 105 °C was used to determine the constant weight [53]. Fat content was determined using the Soxhlet method using petroleum ether extraction, and the results were expressed as a percentage of raw meat [54]. The Kjeldahl method was used to determine the total protein content [55].

#### 2.6.2. Water Holding Capacity

Water holding capacity was determined by the method of Grau-Hamm [56], as modified by Pohja and Niinivaara [57]. A sample of about 1 g of meat was pressed with a 5 kg weight for 5 min; the parameter was determined by the difference in the weight of the sample.

#### 2.6.3. Drip Loss

Drip loss in % was measured by the difference in weight of samples (a slice of fresh meat weighing about 50 g) before and after 48 h of storage at 4 °C in plastic bags.

#### 2.6.4. Cooking Loss

Cooking loss was analyzed according to the Baryłko-Pikielna method [58]. Samples of fresh meat with an initial weight of about 300 g were placed into plastic bags and then heated in water until the internal temperature of the meat reached 70 °C. After removal from water, the samples were cooled and then weighed again. The result was calculated based on the difference in weight expressed as a percentage to the raw sample.

#### 2.6.5. Cutting Force (Texture)

The cutting force of cooked meat samples (cooking condition in Section 2.6.4) was measured using a Zwick Roell instrument (Zwick Roell, Ulm, Germany). The cutting force measurements were made with the Warner-Bratzler V-shaped blade attached to the TA.XT Plus Texture Analyser (Stable 136 Micro Systems, Godalming, UK; test speed 2 mm/s; distance 20 mm; force 20 g). The muscle samples were cooled (to 4 °C) and stored overnight prior to cutting force determination. The Zwick Roell Z0.5 was used with a load of 500 kN and a measuring speed of 100 mm/min. The cutting force was measured parallel to the muscle fibers on cooked meat samples prepared by cutting cylindrical samples (25.4 mm radius) from the meat.

#### 2.6.6. pH Measurements

The pH was measured using an Elmetron CP- 551 pH-meter with the ERH-12-6N electrode (Elmetron, Zabrze, Poland) after calibration with pH = 4 and pH = 7 buffers. The electrode was embedded in the 3 cm thick cut (*Longissimus thoracis et lumborum*) at a depth of 1.5 cm. Three measurements were made for each meat cut, each time placing the electrode in a different part of the cross-section of a loin.

#### 2.6.7. Marbling Score

Marbling was assessed according to the standard on a 5-point scale with the following explanations: 1 point—invisible marbling, 2 points—weakly marbling, 3 points—moderate marbling, 4 points—strong marbling, 5 points—very strong marbling.

#### 2.6.8. Color Analysis

Instrumental color measurements were carried out using a CM-5 Konica Minolta spectrophotometer (Konica, Tokyo, Japan) with SCE (Specular Component Included) mode, 30 mm diameter measurement area, D65 illuminant, and 10° standard observer. Three automatic measurements were made on the 3 cm thick chops, and the mean values of the color coordinates in the CIE L*a*b* color space (lightness, L*; redness, a*; yellowness, b*; chroma, C and hue angle, h) were obtained. The redness index was calculated as the ratio of a*/b*, and reflectance spectra (360 nm–740 nm) were monitored. Color measurements on the first day of storage (i.e., 48 h post-mortem) were performed on freshly cut chops to monitor DeoMb, which is easily oxygenated in the presence of oxygen. The samples were then packed into the propylene bags (CRYOVAC^®^ packaging-safe bags) and stored at 4 °C in the dark for color measurements on days 4, 7, and 10 of storage.

#### 2.6.9. Determination of Surface Myoglobins

Determination of surface myoglobin forms was carried out from reflectance spectra using a CM-5 Konica Minolta spectrophotometer in a range of 360–740 nm, as previously described [59,60]. The reflectance readings were converted to reflex attenuation as log (reflectance) and used in the equations of Krzywicki [59] to determine the percentage contribution of MetMb, OxyMb, and DeoMb.

#### 2.6.10. Determination of Total Myoglobin (Mb)

The determination of total myoglobin (Mb) content in the meat was based on the method of Krzywicki [59] and Trout [60]. Briefly, 5 g of meat was mixed with 40 mmol phosphate buffer pH 6.8 in a 1:5 (*m*/*v*) ratio. The samples were homogenized using an Ultra-Turrax T25 homogenizer (IKA, Staufen, Germany) (16,000 rpm) for 30 s and centrifuged at 3500 rpm at 4 °C for 30 min. After filtration, absorbance was measured at 525 nm and 700 nm. The Mb concentration was calculated using Equation (1):

(1) $$\mathrm{Mb}=(A_{525}+A_{700})/\varepsilon \;\times M_{Mb}\times \mathrm{dilution}\;\mathrm{factor}$$

where ε is the millimolar extinction coefficient for Mb at 525 nm and *M_Mb_* is the average molecular mass of Mb in kDa.

#### 2.6.11. Thiobarbituric Acid Reagent Substances (TBARS)

Lipid oxidation of meat was evaluated based on the method of Mielnik et al. [61], with some modifications [62,63]. The procedure was described in detail in our previous study [62]. To avoid TBARS value overestimation resulting from interfering compounds (flavonoids) present in the sample, 5 mL of the samples was mixed with 5 mL of distilled water (no TBA), and the absorbance was measured at 532 nm [63]. The TBARS values were calculated from the standard curve of MDA (malonodialdehyde) obtained from the oxidation of 1,1,3,3-tetramethoxypropane. When calculating the TBARS values, the final absorbance values were corrected by the absorbance values of the samples without TBA. The final results were expressed in mg of MDA per kg of meat.

#### 2.6.12. Antioxidative Status (DPPH Antioxidant Activity, Enzyme Activity and Total Glutathione)

The antioxidant activity of meat was determined using the DPPH method [64]. Briefly, 0.05 g of meat was mixed with 5 mL of 0.04% DPPH in methanol and kept at ambient temperature for 30 min. Then, samples were centrifuged at 1400 rpm for 10 min, and the absorbance was measured at 515 nm using methanol as a blank sample.

The content of low-molecular-weight antioxidants such as total glutathione (GSH + GSSG) and the activity of the antioxidant enzymes such as CAT (catalase) and SOD (superoxide dismutase) in meat were measured spectrophotometrically according to the established procedures described in detail in the literature [65,66,67].

#### 2.6.13. Sensory Analysis

The sensory evaluation of cooked meat was performed by a team (panel of judges with proven sensory sensitivity) of 5 selected and trained panelists, consisting of employees of the Prof. Wacław Dąbrowski Institute of Agricultural and Food Biotechnology in Warsaw, using a five-point hedonic scale according to the methodology described in ISO (International Standard Organization) standards [68,69,70]. The assessors participated in three specific one-hour training sessions before the test sessions following ISO standards [68,69,70]. The assessment was carried out in daylight at room temperature, with a maximum of 10 samples per session. Three test sessions were performed. Samples were coded with random numbers and given in randomized duplicates. The panelists used a list of qualitative descriptors established at a special session (based on AMSA guidelines [71]) for the sensory attributes: color, aroma, tenderness, juiciness, and palatability. The following grading scale was used: the aroma of pork: 1 point absence (not typical for pork meat, strong deviation from the quality), 5 points intense (typical for pork meat, no deviation from the quality descriptors), tenderness: 1 point very tough, 5 points very tender, juiciness: 1 point very dry, 5 points very juicy, color: 1 point not typical for pork meat (strong deviation from quality descriptors), 5 points typical for pork meat (no deviation from quality descriptors), palatability: 1 point very poor (not typical for pork meat, strong deviation from the quality descriptors), 5 points excellent (typical for pork meat, no deviation from the quality descriptors), where 3 points is the threshold for each characteristic.

### 2.7. Antioxidative Status of Blood and Liver Tissues

#### 2.7.1. Determination of Enzyme Activities and Total Glutathione

The content of GSH + GSSG and the activities of CAT and SOD in blood and liver were measured as described for meat samples in Section 2.6.12. The antioxidant activity of blood expressed as ferric-reducing antioxidant power (FRAP) was determined using Benzie and Strain method [50].

#### 2.7.2. Lipid Peroxidation

The lipid peroxidation product (MDA) in blood and liver was determined using the methods described by Esterbauer and Cheeseman [72].

### 2.8. Statistical Analysis

Statistical analyses were performed using Statistica 13.3 software (StatSoft, Tulsa, OK, USA). The Student t-test was used to check the effect of dietary supplementation of pigs with phenolics on tested parameters of fresh meat. Repeated measures ANOVA was performed for color and pH measurements to monitor the effect of time and sample on the parameters tested. The uniformity of variance was tested using the Cochran–Bartley test, and Mauchly’s test was used to test the assumption of sphericity. The Greenhouse–Geiser correction was applied for lack of sphericity. The correlation between the parameters was shown using r Pearson’s correlation coefficients. Principal component analysis (PCA) was used to gain further insight into meat quality.

Multivariate linear regression (MLR) analysis was used to show the effect of time and diet on pH, TBARS values, the Mb content, the antioxidant activity, and surface myoglobins. To compare the rates (slope of the regression equation) of parameter changes with time between the control and treated samples (showing the effect of diet), an equation was used:

(2) $$y=\beta_{0}+\beta_{1}t+\beta_{2}D+\beta_{3}Dt+\varepsilon ,$$

where: *y*—variable value (pH, TBARS, Mb, DPPH, DeoMb, MetMb); *β*_0_—intercept for control; *β*_1_—regression coefficient for control sample; *β*_2–3_—regression coefficient for dummy variables; *t*—time; *D*—first dummy variable for diet (0 or 1); *Dt*—second dummy variable (product of the first dummy variable and time); *ε*—standard estimation error.

For each parameter, comparisons were made between the regression coefficients of the control and treated sample (P-suppl.), introducing two dummy variables as predictors in the regression analysis. The first dummy variable was coded as 0 for control and 1 for the P-suppl. sample, and the second dummy variable was the product of the first dummy variable and time. Equation (2) was modified as followed:

(3) $$when\;D=0,\;y=\beta_{0}+\beta_{1}t+\varepsilon ,$$

(4) $$when\;D=1,\;y={(\beta }_{0}+\beta_{2})+{(\beta }_{1}+\beta_{3})t+\varepsilon ,$$

Thus, the null hypothesis that the two slopes are equal (no effect of diet) is equivalent to *β*_3_ = 0. The *t*-test was used to evaluate the significance of the regression coefficients for dummy variables. Discrimination coefficients (R^2^) were used to assess curve fitting and ANOVA to evaluate the significance of linear regression. The level of significance for all analyses was set at α = 0.05.

## 3. Results

### 3.1. The Antioxidant Activity and Phenolic Content of the Supplement

The plant-based supplement with phenolics (P-suppl.) for animal nutrition shows a potent antioxidant activity, expressed through different methods, namely 2430.3 ± 308.27 μmol/g for TEAC, 1949.1 ± 90.06 μmol/g for DPPH and 1153.7 ± 52.76 μmol/g for FRAP. The antioxidant activity is the result of the high phenolic content since the TPC value is 150.05 ± 3.5 mg GAE/g, including TFC = 55.67 ± 1.32 mg QE/g. The total phenolic content was about 15% of the P-suppl. mass.

### 3.2. Fresh Meat Quality Attributes

The quality parameters of fresh meat are shown in Table 2.

There were no statistically significant differences between the groups in the chemical composition of meat and other physicochemical properties (*p* > 0.05). Although the drip loss was lower in meat of the pigs fed a phenolic-rich diet (5.41%) than in those from the control group (6%), the differences were statistically insignificant due to high variability. The cutting force was higher in the P-suppl.-treated group than in the control group, which is related to higher pH values (pH 5.88 and 5.84 for treated and control group, respectively), but, again, the differences were not significant.

### 3.3. Color Profile of Fresh Meat

As demonstrated in Table 3, a statistically significant effect of the diet was observed in terms of the a*, h, C, RI, and Mb parameters.

Following the supplementation of the diet with the phenolic-rich additive, a significantly higher redness was observed in the meat muscles than in the control group. Significantly higher a* and RI values and significantly lower h values for the experimental group were observed in comparison to the control sample. The Mb content was observed to be slightly higher in the treated group than in the control. The treated samples are, therefore, slightly darker than the control sample, with the difference reflected in the L* values.

**Figure 1:**
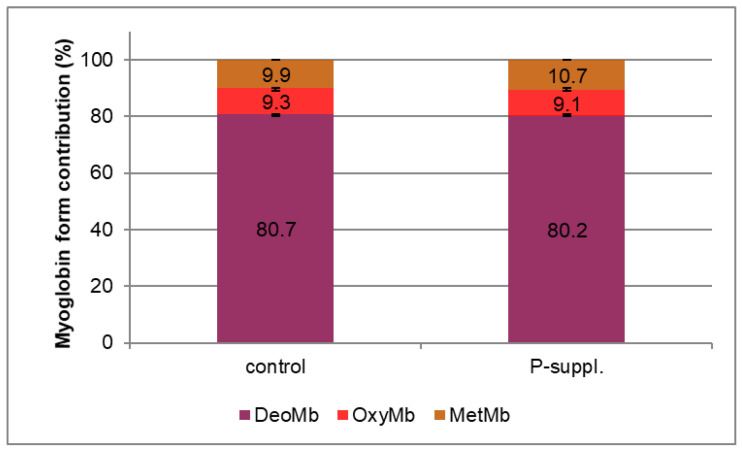
Contribution (%) of various myoglobin forms. DeoMb, deoxymyoglobin; OxyMb, oxymyoglo-bin; MetMb, metmyoglobin. Each profile represents the mean values (n = 12).

The contribution of meat pigments—various myoglobin forms—in the tested samples is shown in Figure 1. The supplementation of the porcine diet with polyphenols did not result in significant changes in the myoglobin forms. The predominant form was DeoMb, the purplish-red-colored pigment, which is characteristic for the fresh-cut meat. The proportion of bright-red OxyMb was approximately 9% in both samples. Similarly, the proportion of brown MetMb was approximately 10%, with no significant differences between the control and treated samples (*p* = 0.65).

**Figure 2:**
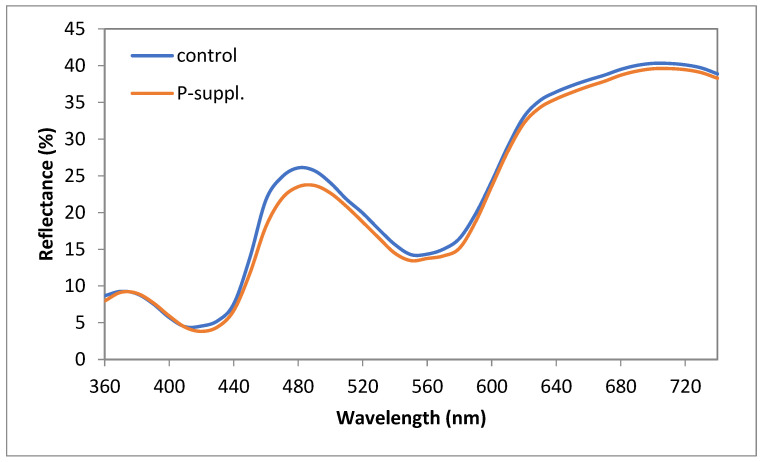
Reflectance spectra of fresh meat. Each profile represents the mean values (n = 12).

When analyzing the reflectance spectra of the control and treated samples (Figure 2), a slight difference was observed in the shape of the reflectance curve profile.

From the reflectance profile of the control sample, the characteristic spectra of DeoMb could be observed, with the minimum reflectance occurring in the 540–580 nm region. In the case of the treated sample, a slight decrease in reflectance values was observed within a range of 410 to 580 nm, accompanied by a shift in the maximum reflectance peak from 480 nm to 490 nm when compared to the control. This finding could indicate OxyMb formation.

Furthermore, the ratio of 630 nm to 580 nm yielded a higher value for the treated sample (2.27) than for the control (2.14). A higher ratio indicated a greater redness, which could be attributed to either OxyMb or DeoMb (it is not specific for OxyMb, since DeoMb is more red than MetMb at 630 nm [19]). This finding is consistent with the color parameters presented in Table 3.

### 3.4. Oxidative Stability Parameters of Fresh Meat

**Figure 3:**
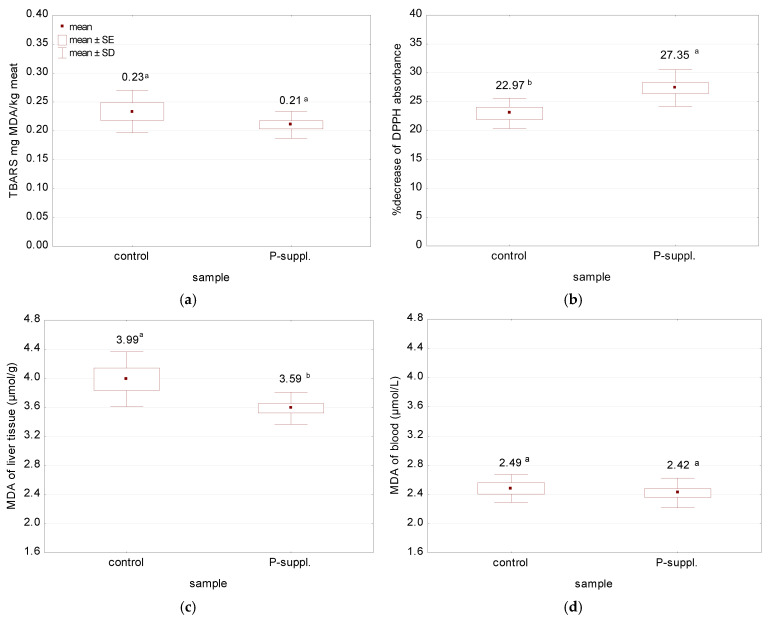
Effect of diet on: (**a**) lipid oxidation of meat by TBARS; (*p* = 0.132); (**b**) the antioxidant activity of meat measured by DPPH method (*p* = 0.011); (**c**) lipid peroxidation of the liver tissue (*p* = 0.011); (**d**) lipid peroxidation of the blood (*p* = 0.535), where *p* ≤ 0.05 indicates a statistically significant effect of the diet. ^a,b^ the same lower superscript letters indicate a lack of statistically significant differences between samples (within tested parameters). TBARS, thiobarbituric acid reactive substances; MDA, malonodialdehyde; DPPH, 1,1’-diphenyl-2-picrylhydrazyl; SE, standard error; SD. Standard deviation.

It has been previously reported that the addition of a phenolic-rich supplement to the animal diet may result in an increase in the oxidative stability of meat and other tissues. Consequently, the extent of lipid peroxidation was expressed as MDA content; the activities of antioxidant enzymes (CAT, SOD) and total glutathione (GSH + GSSG) were measured in the blood, liver, and meat samples. Furthermore, the antioxidant activity of the meat was assessed using the DPPH method, while the antioxidant potential of the blood was determined using the FRAP method. The results are illustrated in Figure 3 and Table 4.

A detailed analysis of the data presented in Figure 3 revealed a significant effect of dietary supplementation with phenolics on the antioxidant activity of meat (Figure 3b) and the MDA content of the liver (Figure 3c). The antioxidant activity of the meat was higher in the phenolic-supplemented group than in the control group, which was reflected by the higher percentage decrease in DPPH absorbance. However, the diet did not influence the lipid peroxidation of meat, although the TBARS values of the treated sample were lower than for the control one (Figure 3a). Furthermore, no statistically significant correlation was found between TBARS and the antioxidant activity of meat (r = −0.158, *p* = 0.513). The level of lipid peroxidation in liver tissues was significantly higher in the control sample in comparison to the P-suppl. sample, with MDA contents of 4.46 and 3.89 μmol/g, respectively (Figure 3c). No significant differences in MDA content were observed between the control and treated groups in the blood samples (Figure 3d).

The antioxidative status of samples is presented in Table 4. In blood and liver tissues, CAT, SOD activities, and GSH + GSSG content increased significantly in the phenolic-supplemented groups (*p* ≤ 0.05). The supplementation was also observed to enhance the antioxidant activity of the blood, as evidenced by an increase in FRAP values in the treated samples. In the meat of P-suppl. pigs, the SOD activity and GSH + GSSG content were significantly higher than in the control group, but the CAT activity was lower. These parameters were highly correlated with the DPPH antioxidant activity (with the correlation coefficients r = 0.529 and *p* = 0.024 for SOD; r = 0.517 and *p* = 0.024 for GSH + GSSG; and r = −0.539 and *p* = 0.021 for CAT).

### 3.5. Sensory Analysis of Fresh Meat

**Figure 4:**
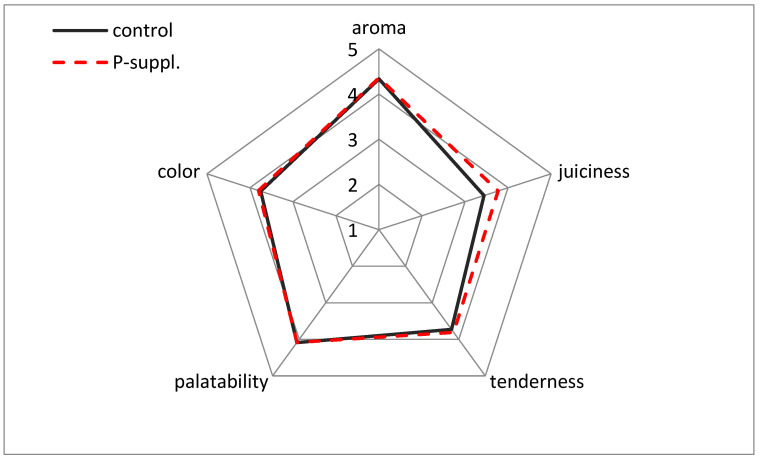
Sensory analysis profile of the control and phenolic-supplemented meat samples.

The meat was characterized by high scores on a five-point scale. The results are illustrated in Figure 4.

The lowest scores were for juiciness (3.44 and 3.77 for the control and P-suppl. group, respectively), while the highest were for aroma (4.34 and 4.36 for the control and P-suppl. group, respectively). The supplementation of the pigs’ diet with phenolics was found to significantly increase the juiciness of the meat (*p* = 0.007). No significant effect of the diet was observed for the other sensory attributes.

### 3.6. Multivariate Analysis of Fresh Meat Quality Parameters

**Figure 5:**
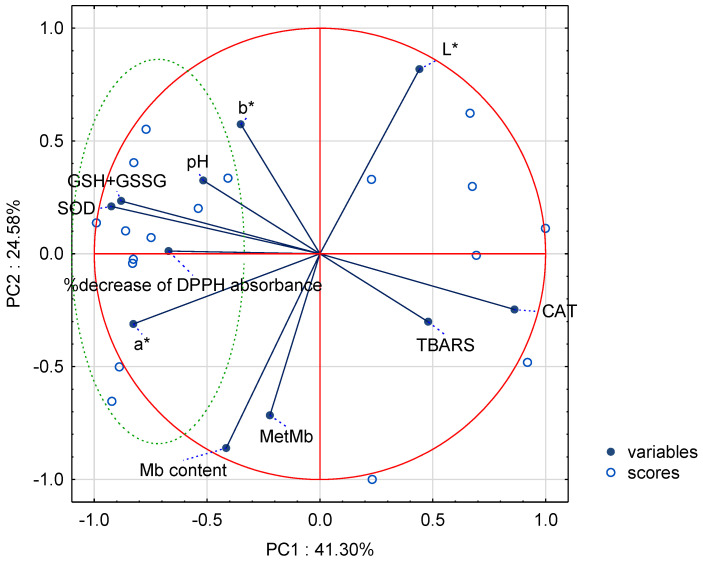
PCA results (biplot) of fresh meat for the variables and scores.

To put more insight into the dataset, PCA was performed. To this end, the selection of variables (according to their importance) for multivariate analysis was performed using the data mining tool in Statistica 13.3 software. The four principal components (PCs) had eigenvalues above 1, with the first two explaining 66% of the total variability. Based on the loaded values, showing the correlations between components and variables, it could be stated that the a* (−0.83), CAT (0.86), SOD (−0.92), and GSH + GSSG content (−0.88) of the meat determined the sample distribution along PC1 (Figure 5).

The L*, Mb content, and MetMb variables distributed the samples along PC2 with correlation coefficients of 0.82, −0.86, and −0.72, respectively. Figure 5 also illustrates the projection of the scores on the factor plane. PC1 enabled us to separate the P-suppl. samples (on the left of PC1 axis, in a green circle), with the highest SOD activity, GSH + GSSG content, and DPPH antioxidant activity, from the control group (with the highest TBARS and CAT activity values).

### 3.7. Effect of Storage Time on Meat Quality

**Figure 6:**
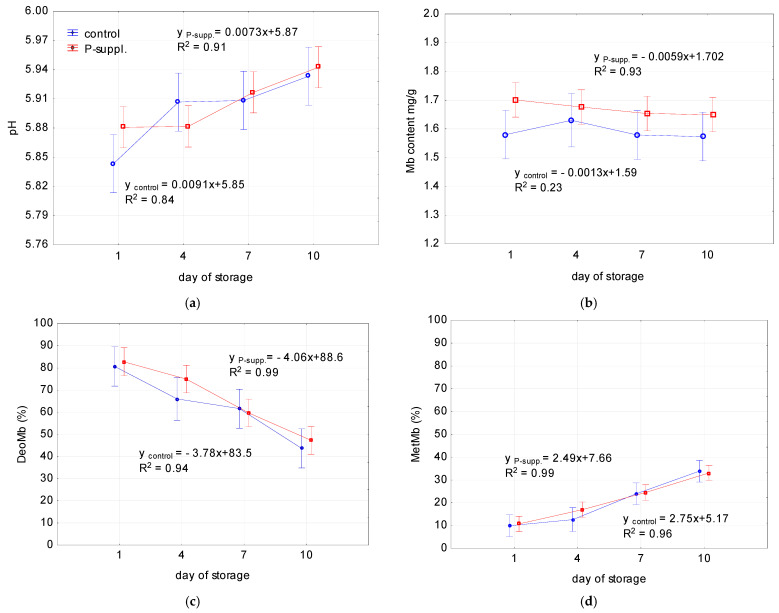

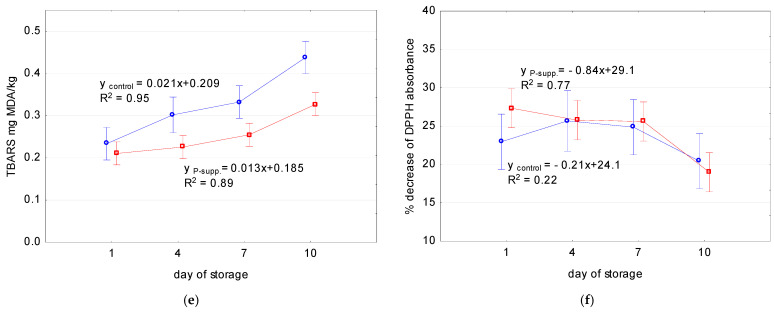
Effect of time on: (**a**) pH; (**b**) myoglobin (Mb) content; (**c**) deoxymyoglobin (DeoMb); (**d**) metmyoglobin (MetMb) (**e**) lipid peroxidation (TBARS); (**f**) DPPH antioxidant activity of control (blue) and treated sample (red). R^2^, determination coefficient.

The storage quality of fresh meat is crucial for consumers, and, thus, the pH, color, and pigment content, as well as TBARS values and the DPPH antioxidant activity of meat, were monitored over time. The results are presented in Table 5 and Figure 6.

A statistically significant effect of time was observed for all the parameters tested, with the exception of the Mb content and the antioxidant activity of the control samples (Table 6). The lipid peroxidation of meat, as indicated by the TBARS values, was also influenced by dietary supplementation, which was proved by the significant differences between the control and P-suppl. slopes for the linear regression models (Table 6).

A significant increase in L*, a*, b*, C*, and RI was observed during the storage period. The Abs525 nm value (reflecting meat pigmentation) and the h value both demonstrated a significant decline over time.

As illustrated in Figure 6a, the pH values of both samples increased over time, reaching a value of about 5.94 on the 10th day. The Mb content ranged from 1.57 mg/g to 1.70 mg/g, with the phenolic-supplemented group exhibiting a higher level (1.65–1.70 mg/g) than the control group (1.57–1.58 mg/g). However, the parameter remained unaffected by time and sample (Figure 6b). The percentage of DeoMb decreased, while the percentage of MetMb increased with time. No statistically significant differences were observed between the control and supplemented groups (Figure 6c,d). The extent of lipid peroxidation in meat (Figure 6e) increased during storage, reaching the maximum TBARS values on the 10th day (0.44 and 0.33 mg MDA/kg for control and P-suppl. samples, respectively). The slope of the plot for TBARS with time of storage was significantly higher in the control sample than in the P-suppl. group (Table 6) indicating a significant effect of diet on TBARS values. Although the DPPH antioxidant activity was higher in the phenolic-supplemented sample on the first day of storage, the differences between the control and dietary-modified group were no longer observed on other days of storage (Figure 6f). The antioxidant activity of the control and P-suppl. samples decreased with time, and the decrease was most pronounced between the 7th and 10th days of storage.

## 4. Discussion

Several studies have investigated the effect of natural antioxidant supplementation of finishing pig diets on meat quality [30,31,34,35,73]. However, the data still give inconclusive results. Plant-based supplements for animal nutrition, which are available on the market, aim at increasing or maintaining feed efficiency and, at the same time, counteracting oxidative stress in meat. They are claimed to contain a mixture of various naturally occurring antioxidant active compounds, including tocopherols, phenolic acids, and polyphenols, and to improve meat quality due to an inhibition or reduction in oxidative stress. Thus, this study investigated the effect of a commercially available phenolic supplement in the finishing diet (0.1%) of pigs on meat quality, expressed as proximate composition, pH, color, myoglobin forms, and the antioxidative status, as well as the oxidative stability during storage.

As reported by others, the proximate composition of meat [30,31] and marbling scores [31,34,35] were unaffected by the dietary modification with natural extracts. A concentration-dependent effect was previously observed, as high dietary doses of natural antioxidants increased crude protein in pork [31]. The marbling score is related to the eating quality of meat and is, therefore, often correlated with sensory characteristics, such as tenderness or juiciness [74]. In our study, there was no correlation between the marbling score and juiciness or tenderness.

The pH of meat indicates its quality, affecting the color, water retention, tenderness, and sensory properties [34]. In this study, the differences between the pH values of the two groups tested were not statistically significant. Our findings are consistent with those of O’Grady et al. [37], who showed that a diet containing grape seed extract and bearberry did not affect the pH values, or with those of Cheng et al. [35], who reported no effect of oregano essential oil supplementation on pH. However, others reported a significant effect of animal nutrition with natural antioxidants on the pH of meat [31]. Dietary grape seed supplementation of finishing pigs resulted in an increase in pH [31], which was related to the effect of supplementation on the muscles’ glycolytic potential and lactase content [31].

Cooking loss and drip loss are of great importance for the water retention capacity of the muscles, which is closely related to pH and, thus, to meat quality, especially sensory characteristics (tenderness) [34]. In particular, as indicated by Zeng et al. [34], a decrease in pH at 45 min is associated with an increase in drip loss, which negatively affects meat quality. Although the values of cooking loss and drip loss were lower in this study after phenolic supplementation than in the control sample, the differences were not statistically significant. Cooking loss and drip loss did not correlate with water holding capacity (r = −0.31 and r = 0.14 for the correlation with cooking loss and drip loss, respectively), either with the cutting force (r = −0.31 and r = 0.06, respectively) or pH 45 min (r = −0.04 and r = −0.36, respectively). Our results are in agreement with the results of Xu et al. [31], who reported no effect of grape seed supplementation on cooking loss and drip loss. Others reported that the addition of mulberry leaves to pigs’ diets tended to significantly decrease cooking loss, drip loss, and shear force [34] or the addition of oregano essential oil decreased cooking loss and drip loss [35]. A significant reduction in shear force after supplementation with natural antioxidants was reported by Xu et al. [31]. Interestingly, the lower the drip loss, the higher the DPPH antioxidant activity of meat. r-Pearson’s coefficient for the correlation is r = −0.56 and is statistically significant (*p* = 0.015). The correlation between drip loss and body antioxidative status was also noted after pig diet supplementation with mulberry leaf extract [34] or with oregano oil [35]. This could be an effect of the antioxidant activity of the supplement. The phenolic compounds from the supplement could protect the integrity and, thus, decrease the permeability of cell membranes due to the inhibition of lipid and/or protein oxidation [35].

The color of meat is a very important quality attribute that affects consumers’ decision at purchase and consumers’ perception of meat [18]. In our study, dietary supplementation did not affect the L*, b*, and Abs525 nm of meat, but it increased the a*, C, and RI and decreased the hue angle. This is consistent with the studies of Xu et al. [31], who showed no differences in the lightness and yellowness of meat among the control and grape-seed-supplemented groups, whereas dietary modification with a phenolic-rich supplement increased the a* of pork from 5.7 to 6.85. The observed increase in redness after phenolic supplementation could be an effect of the anthocyanins, the dominant group of flavonoids in the tested supplement, as indicated by the producer. Anthocyanins are plant-derived pigments that give a red to purple-blue color, with a maximum absorbance at 520–550 nm and maximum reflectance at around 580–650 nm. They are found as potent antioxidants among flavonoid compounds. These plant pigments could be present in the intact form in the pig tissues after fasting the animals with the anthocyanin-rich diet, which has been previously reported by Kalt et al. [75]. As well as giving a specific color to the meat samples, they also act as antioxidants and protect myoglobin from oxidation, which affects the color of the meat. Scerra et al. [30] reported that feeding pigs with the bergamot by-product did not affect color parameters (lightness, redness, yellowness, chroma, and hue angle). Similarly, the addition of mulberry leaf extract to the pig diet had no effect on the color parameters [34]. Introducing tomato pomace to the finishing pig diet did not influence the chromatic color attributes of meat (a* and b*) but significantly reduced the L* values from 53.5 to 50.3 [33].

Lightness is an achromatic color attribute, and the lightness of meat depends on the total amount of light absorbed (achromatically and chromatically) or reflected from its surface [76]. Thus, the lightness of meat depends on the content of pigments in tissues and the meat surface and structure [76]. It was previously observed that lightness is inversely correlated with the content of Mb [77]. In our study, L* was significantly correlated with redness (a* and RI parameters), hue angle, and Mb content. The higher the Mb content of fresh meat, the higher the redness and the lower the lightness. The correlation coefficients were −0.53 (between L* and a*), −0.69 (L* and RI), 0.69 (L* and h), and −0.99 (L* and Mb content). In this study, the L* of fresh meat was also inversely related to MetMb (r = −0.43, *p* = 0.075), which is in agreement with the studies of Biondi et al. [33], who reported that MetMb formation decreased the lightness of meat. However, others did show that MetMb formation on the meat surface increased lightness, albeit this increase gives the impression of a lightness reduction due to changes in the hue angle from red to dark red/brown [77].

The color of meat is determined by the changes in the myoglobin forms. The interconversion of myoglobin from the ferrous forms (DeoMb and OxyMb) to the ferric form (MetMb) is often correlated with lipid peroxidation [78]. Thus, lipid oxidative changes could cause color alterations, mostly meat discoloration. Significant correlations between the a* and Mb content (r = 0.55) as well as the a* and MetMb (r = 0.58) were observed. Surprisingly, in our study, no other correlations were noticed between the color parameters of fresh meat and surface myoglobins or with TBARS values. Oxidized MetMb contributed to only 10% of total Mb forms in our study. The predominant form of myoglobin in the pork samples was DeoMb (around 80%), indicating a very good quality of meat and reflecting its freshness [19]. According to the survey on consumers’ expectations at purchase, 29% of them preferred the light-red color of meat [18].

The lipid oxidation of fresh pork meat, expressed as TBARS values, was unaffected by dietary supplementation of pigs with phenolics, although a significant increase in the DPPH antioxidant activity of meat was obtained after phenolic addition to the pig diet. The lack of effect of adding natural antioxidants to animal diets on TBARS has already been reported in other studies [30,33,37]. In contrast, a significant decrease in the TBARS values was also noted after dietary modification with antioxidants [31]. The supplementation of pig feed with antioxidants to improve meat quality is highly dependent on many factors and is not always effective. It depends on the bioavailability and metabolism [36]. As reported in a review [36], only 5% to 10% of ingested phenolic compounds can be absorbed in the intestine and undergo further biotransformation in enterocytes and, in hepatocytes, to form methylated, glucuronidated, or sulphated conjugates. These conjugate metabolites enter the bloodstream and are distributed through the body and, finally, excreted in the urine [36]. To monitor the uptake and distribution of antioxidants in the animal body, the level of lipid peroxidation was investigated in the liver tissues and blood. The phenolic-rich diet resulted in a significant decrease in the MDA content in the liver, but the effect was no longer observed in the blood. This could be due to the low t1/2 of the flavonoid compounds in the blood plasma, which is less than 2 h, and the accumulation of phenolic compounds, even in their native forms, in the liver tissues [75]. O’Grady et al. [37] showed no effect of dietary modification with natural antioxidants on the MDA content in liver tissues.

Living organisms have several endogenous antioxidant systems, which are the most important defense line to protect the body against oxidative damage. These include antioxidant active enzymes, such as SOD, CAT, and low-molecular-weight antioxidants such as GSH + GSSG. These systems can be activated by various bioactive substances such as phenolics in different ways [31,79]. The signaling pathway through the Nrf2 is crucial for the expression of genes coding the antioxidant enzymes such as CAT or SOD or phase II biotransformation enzymes [79]. Interestingly, this can also be mediated by the pro-oxidant activity of polyphenols with the phenolic quinone metabolites as the intermediates in the induction of the gene expression response [79]. Further studies are required to explain this mechanism. In this study, the dietary administration of antioxidants increased the activities of CAT, SOD, and GSH + GSSG content in blood and liver tissues and the activity of SOD and GSH + GSSG content in meat. The FRAP antioxidant activity of the blood was positively correlated with SOD (r = 0.64), CAT (r = 0.75) activities and GSH + GSSG content (r = 0.48) in blood and SOD activity and GSH + GSSG in meat (r = 0.82 and r = 0.79, respectively) and the liver (r = 0.86 and r = 0.67, respectively). Our studies are consistent with others [31,32] reporting significant effects of dietary antioxidants on SOD and CAT activities.

The juiciness score for the treated group was significantly higher than for the control group, which is in contrast to the results obtained by others [35,37]. The sensory attribute is highly correlated with the DPPH antioxidant activity (r = −0.50), CAT activity (r = −0.57), SOD activity (r = 0.59), and total glutathione (r = 0.66). It was previously observed that lower juiciness is caused by protein oxidation in meat muscles [13]. The antioxidants could protect proteins from oxidation, maintaining their functionality and, thus, affecting juiciness [13]. Although a significant effect of a phenolic-rich diet was observed on the physicochemical parameters of meat, the sensory analysis did not show any differences between the groups for color, aroma, palatability, and tenderness attributes. This could be an effect of testing the fresh meat (48 h after slaughter). Thus, the meat quality was monitored with storage time at 4 °C for 10 days.

A significant effect of time was observed for pH, color, myoglobin form contribution, lipid peroxidation, and the DPPH antioxidant activity. This is in agreement with previously reported results [30,37]. A significant effect of dietary modification was only noted for TBARS values, indicating that the addition of natural antioxidants to the animal diet increased the oxidative stability of the meat during storage. It is also worth mentioning that the TBARS values are below the threshold proposed for the rancidity (from 0.5 to 1 mg MDA/kg of meat), indicating high meat quality [33,37].

## 5. Conclusions

In conclusion, dietary supplementation with a plant-based phenolic supplement (0.1%) improved the color and antioxidant status of meat. The separate distribution of the treated samples on the principal component plane indicated a significant effect of the diet. Based on the PCA results, it could be concluded that the color, oxidative changes (lipids and pigments), and antioxidant status of meat were interrelated and made a complex process. Although the TBARS values of 48 h post-mortem meat samples were not affected by the diet, the antioxidant effect of phenolics was observed during storage. These findings provide a new nutritional approach for the production of high-quality pork for human consumption and indicate a significant effect of phenolic supplementation on the color and antioxidant status of fresh meat, as well as an improvement in the oxidative stability of lipids during meat storage. However, further studies are required to explain the mechanism of antioxidant enzyme activation (CAT, SOD activity) and increase in GSH + GSSG content by the phenolic compounds of the tested supplement.

## Figures and Tables

**Table 1:** Composition and nutritional value of the basal diet in two phases (grower and finisher).

Item	Grower	Finisher	
	Control and P-Suppl. ^1^	Control	P-Suppl.
Composition (kg/ton)			
Cereals and cereal grain co-products—barley, corn, wheat, wheat bran	800.3	826.4	825.4
Soybean meal and rapeseed meal	160.0	140.0	
Limestone	14.7	12.0	
Oil	13.0	13.0	
NaCl	4.0	4.0	
HCL-lysine	3.5	2.4	
Premix 0.2% ^2^	2.0	2.0	
1-calcium phosphate	1.8	0.0	
DL-methionine	0.3	0.0	
L-threonine	0.2	0.0	
Phytase—5000 FTU	0.1	0.1	
Endo-1,4-beta-xylanase—15,000 EPU	0.1	0.1	
P-suppl. ^1^	0.0	0.0	1.0
Nutritional value (g/kg)			
Crude protein	162.1	148.7	149.1
Crude fat	36.4	34.1	33.8
Crude fibre	50.8	54.1	54.4
Crude ash	53.41	48.7	49.1
Dry matter	889.0	990.3	991.0
Ca	8.0	6.4	6.4
P	5.4	5.2	5.1
Lysine	10.2	8.6	8.6
Methionine	3.0	2.5	2.5

^1^ P-suppl.-natural plant-based supplemented diet with phenolics; ^2^ Premix—the mineral and vitamin premix contained the following amounts of components per 1 kg: vitamin A—10,833 mg/kg, vitamin D3—25 mg/kg, vitamin E—50,000 mg/kg, vitamin K3—2000 mg/kg, vitamin B1—1000 mg/kg, vitamin B2—2000 mg/kg, vitamin B6—1500 mg/kg, vitamin B12—15 mg/kg, pantothenic acid—1500 mg, nicotinic acid—1000 mg/kg, biotin—50 mg/kg, folic acid—750 mg/kg, Fe—50,000 mg/kg, Mn—37,000 mg/kg, Zn—50,000 mg/kg, Cu—10,000 mg/kg, I—750 mg/kg, and Se—200 mg/kg.

**Table 2:** Fresh meat quality attributes.

Quality Attributes	Control	P-Suppl.	SE	*p* *
Fat (%)	1.66 ± 0.70	1.66 ± 0.63	0.149	0.996
Protein (%)	24.85 ± 1.17	24.92 ± 0.89	0.226	0.893
Water (%)	72.53 ± 0.93	72.47 ± 1.11	0.253	0.905
pH 45 min	6.31 ± 0.32	6.50 ± 0.24	0.065	0.195
pH 24 h	5.50 ± 0.06	5.50 ± 0.11	0.022	0.944
pH ^1^	5.84 ± 0.05	5.88 ± 0.06	0.058	0.204
Water holding capacity (%)	2.34 ± 0.93	2.34 ± 1.20	0.257	0.998
Drip loss (%)	6.00 ± 1.32	5.41 ± 1.71	0.371	0.471
Cooking loss (%)	32.45 ± 3.20	31.22 ± 5.90	1.199	0.644
Cutting force (N/m^2^)	183.42 ± 44.34	205.65 ± 68.20	14.35	0.482
Marbling score	1.50 ± 0.25	1.47 ± 0.32	0.069	0.829

SE, standard error calculated for all results; P-suppl., polyphenol supplemented diet. ^1^ pH value after 48 h post-mortem. * *p* ≤ 0.05 indicates a statistically significant effect of the treatment.

**Table 3:** Color parameters of fresh meat.

Color Parameter	Control	P-Suppl.	SE	*p* *
L*	51.69 ± 3.03	50.53 ± 2.44	0.61	0.393
a*	4.52 ± 0.54	6.15 ± 1.16	0.30	0.005
b*	12.40 ± 1.02	13.07 ± 0.86	0.22	0.160
h (°)	69.91 ± 3.02	64.97 ± 4.15	1.04	0.020
C	13.21 ± 0.93	14.48 ± 1.03	0.27	0.021
RI (a*/b*)	0.37 ± 0.06	0.47 ± 0.09	0.02	0.021
Abs 525 nm	0.73 ± 0.07	0.76 ± 0.05	0.01	0.452
Mb content (mg/g)	1.58 ± 0.08	1.70 ± 0.07	0.03	0.012

L*, lightness; a*, redness; b*, yellowness; h, hue angle; C, chroma; RI, redness index; Mb content, myoglobin content; ABS 525 nm; absorbance at 525 nm; SE, standard error calculated for all results. * *p* ≤ 0.05 indicates a statistically significant effect of the diet.

**Table 4:** Antioxidant status-related parameters of blood, liver and meat of pigs.

Homogenate	Oxidative Status	Control	P-Suppl.	SE	*p* *
blood	CAT (U/mL)	2.46 ± 0.17	3.06 ± 0.17	0.078	0.000
	SOD (U/mL)	14.49 ± 1.18	17.83 ± 1.72	0.525	0.001
	GSH + GSSG (μmol/L)	0.58 ± 0.04	0.68 ± 0.05	0.015	0.001
	FRAP (μmol/L)	9.70 ± 0.99	12.11 ± 0.83	0.342	0.000
liver	CAT (U/g)	101.93 ± 3.82	111.42 ± 9.82	2.209	0.038
	SOD (U/g)	122.76 ± 3.17	135.81 ± 3.65	1.693	0.000
	GSH + GSSG (μmol/g)	0.19 ± 0.012	0.23 ± 0.023	0.007	0.001
meat	CAT (U/g)	22.34 ± 1.23	16.71 ± 0.92	0.685	0.000
	SOD (U/g)	36.81 ± 0.80	43.44 ± 1.67	0.828	0.000
	GSH + GSSG (nmol/g)	239.29 ± 11.45	325.74 ± 13.78	10.329	0.000

* *p* ≤ 0.05 indicates a statistically significant effect of the diet. SE, standard error calculated for all results; MDA, malonodialdehyde; CAT, catalase; SOD, superoxide dismutase; GSH + GSSG, total glutathione; FRAP, ferric reducing ability of plasma.

**Table 5:** Effect of storage time on meat color parameters.

Sample	Days	L*	a*	b*	h (°)	C	RI (a*/b*)	Abs 525 nm
control	1	51.69 ± 3.02 ^a,d^	4.52 ± 0.54 ^a,b^	12.40 ± 1.02 ^a,d,e^	69.91 ± 3.02 ^b,d^	13.21 ± 0.93 ^a,c,d^	0.37 ± 0.06 ^a,b^	0.73 ± 0.07 ^c,d^
	4	52.36 ± 3.19 ^a–d^	5.24 ± 0.62 ^c–e^	12.57 ± 0.94 ^a–g^	67.30 ± 3.21 ^a,c^	13.64 ± 0.82 ^a,c,d^	0.42 ± 0.07 ^c,d^	0.71 ± 0.06 ^a–d^
	7	53.24 ± 2.30 ^b,c,e,f^	5.00 ± 0.69 ^c–e^	13.43 ± 0.89 ^c,h,i^	69.53 ± 3.52 ^b,d^	14.36 ± 0.71 ^b,e,f^	0.38 ± 0.07 ^a,b^	0.71 ± 0.04 ^a,b^
	10	53.62 ± 3.01 ^e,f^	5.81 ± 0.58 ^f–h^	13.04 ± 0.98 ^b–i^	65.90 ± 3.11 ^a,c^	14.29 ± 0.84 ^b,e,f^	0.45 ± 0.07 ^c,d^	0.70 ± 0.06 ^a,b^
P-suppl	1	50.53 ± 2.44 ^a–c^	6.15 ± 1.16 ^e,h^	13.07 ± 0.86 ^e,g,i^	64.97 ± 4.15 ^a,b^	14.48 ± 1.03 ^d,f^	0.47 ± 0.09 ^b,d^	0.76 ± 0.05 ^b,d^
	4	50.94 ± 2.39 ^a–c^	5.14 ± 0.95 ^a,c,f^	12.03 ± 0.75 ^a–c^	66.96 ± 4.20 ^c,d^	13.11 ± 0.76 ^a,b^	0.43 ± 0.09 ^a,c^	0.75 ± 0.05 ^a–d^
	7	51.64 ± 2.50 ^a–c^	5.76 ± 1.01 ^b,d,g^	12.23 ± 0.94 ^a–c^	64.87 ± 4.03 ^a,b^	13.55 ± 1.01 ^c,e^	0.47 ± 0.09 ^b,d^	0.74 ± 0.05 ^a,c^
	10	51.93 ± 2.50 ^d–f^	6.13 ± 0.8 ^e,h^	12.68 ± 0.86 ^d,f,h^	64.25 ± 3.36 ^b,d^	14.10 ± 0.86 ^d,f^	0.49 ± 0.07 ^b,d^	0.74 ± 0.05 ^a,c^

^(a–i)^ The same lower superscript letters indicate lack of statistically significant differences in columns; L*, lightness; a*, redness; b*, yellowness; h, hue angle; C, chroma; RI, redness index.

**Table 6:** Regression analysis with the fixed effect as diet and random effect as pig ID.

Sample	Quality Attributes	Slope	Intercept	R^2^	*p* (ANOVA)
control	pH	0.0091 *	5.85 *	0.84	0.00153
	Mb content (mg/g)	−0.0013	1.59 *	0.23	0.846
	DeoMb	−3.78 *	83.5 *	0.94	0.000001
	MetMb	2.75 *	5.17 *	0.96	0.000001
	TBARS (mg MDA/kg meat)	0.021 *	0.209 *	0.95	0.00001
	% decrease of DPPH absorbance	−0.21	24.13 *	0.22	0.481
P-suppl.	pH	0.0073 *	5.87 *	0.91	0.000013
	Mb content (mg/g)	−0.0059	1.702 *	0.93	0.175
	DeoMb	−4.06 *	88.6 *	0.99	0.000001
	MetMb	2.49 *	7.66 *	0.99	0.000001
	TBARS (mg MDA/kg meat)	0.013 *	0.185 *	0.89	0.000
	% decrease of DPPH absorbance	−0.84 *	29.1 *	0.77	0.00013

* *p* ≤ 0.05 indicate statistically significant regression coefficient; red color indicate that the regression coefficient for slope of P-suppl. sample is significantly different from the corresponding regression coefficient for slope of the control (based on the MLR with dummy variables—fixed effect of diet); Mb, myoglobin; DeoMb, deoxymyoglobin; MetMb, metmyoglobin; TBARS, thiobarbituric acid reactive substances; MDA, malonodialdehyde; DPPH, 1,1′-diphenyl-2-picrylhydrazyl.

## Data Availability

Data are available from the corresponding authors upon reasonable request.
